# 3D and 4D Bioprinting of the Myocardium: Current Approaches, Challenges, and Future Prospects

**DOI:** 10.1155/2018/6497242

**Published:** 2018-04-22

**Authors:** Chin Siang Ong, Lucy Nam, Kingsfield Ong, Aravind Krishnan, Chen Yu Huang, Takuma Fukunishi, Narutoshi Hibino

**Affiliations:** ^1^Division of Cardiac Surgery, Johns Hopkins Hospital, Baltimore, MD, USA; ^2^Division of Cardiology, Johns Hopkins Hospital, Baltimore, MD, USA; ^3^Department of Cardiac, Thoracic and Vascular Surgery, National University Heart Centre, Singapore

## Abstract

3D and 4D bioprinting of the heart are exciting notions in the modern era. However, myocardial bioprinting has proven to be challenging. This review outlines the methods, materials, cell types, issues, challenges, and future prospects in myocardial bioprinting. Advances in 3D bioprinting technology have significantly improved the manufacturing process. While scaffolds have traditionally been utilized, 3D bioprinters, which do not require scaffolds, are increasingly being employed. Improved understanding of the cardiac cellular composition and multiple strategies to tackle the issues of vascularization and viability had led to progress in this field.* In vivo* studies utilizing small animal models have been promising. 4D bioprinting is a new concept that has potential to advance the field of 3D bioprinting further by incorporating the fourth dimension of time. Clinical translation will require multidisciplinary collaboration to tackle the pertinent issues facing this field.

## 1. Introduction

It has been reported that, in the United States, 1 in 7 deaths are attributable to coronary artery disease (CAD) and the estimated incidence of myocardial infarction is 790,000 per year [1]. Despite advances in medical therapy, fibrotic myocardial scar tissue formation after ischemia leading to depressed heart function is generally considered an irreversible process, short of a heart transplant. There have been many attempts at cardiac regeneration, but extensive data suggests transplanted cells, mostly delivered by injection, do not survive in the long term [2]. Cardiac tissue engineering [3] has thus been developed as a promising alternative in the treatment of ischemic heart disease, by cardiac regeneration. One of the newer methods of cardiac tissue engineering is three-dimensional (3D) bioprinting. 3D bioprinting has shown varying degrees of success, where optimization of the tissue engineering process has led to meaningful preclinical applications [4]. Meanwhile, the concept of adding time as an additional dimension to 3D bioprinting has led to the development of a new field of 4D bioprinting. This article aims to summarize the advances and challenges of 3D and 4D bioprinting of the myocardium.

## 2. 3D Bioprinting Methods

3D printing, or additive manufacturing, refers to the creation of physical 3D objects by deposition of material in a successive layered approach, guided by previously specified custom digital designs, and has led to multiple innovations in cardiovascular medicine [5]. This technology has enabled the production of patient-specific 3D objects with varied geometries, sometimes complex, which have clinical applications in medical education, functional flow modelling, procedural planning, and device innovation. In addition, the development of solvent-free, aqueous-based systems have enabled the 3D printing of biomaterials into 3D scaffolds or molds that could be used for transplantation with or without seeded cells [6].

**Figure 1:**
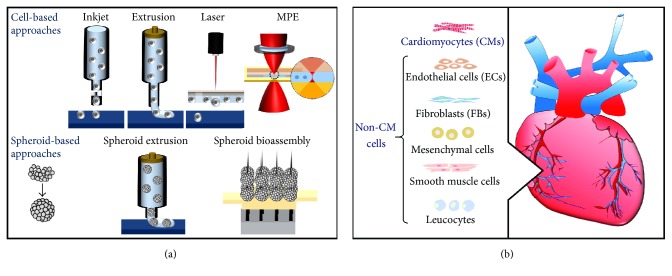
3D bioprinting of the myocardium: methods (a) and cell types (b).

3D bioprinting [4, 7] is a subset of 3D printing and involves the precise positioning of biomaterials and living cells in a layered approach, with spatial control over the arrangement of these functional components. Advances in 3D bioprinting have achieved substantial success in bioengineering of living tissues and organs. Methods of 3D bioprinting include inkjet bioprinting, microextrusion bioprinting, laser-assisted bioprinting, multiphoton excitation (MPE) based fabrication [4, 8], and spheroid-based approaches (Figure 1).

In inkjet bioprinting, thermal and piezoelectric drop-on-demand 3D bioprinters are most frequently utilized [4, 9, 10]. Thermal printing uses heat to create a vapour bubble in the biomaterial containing the cells of interest, which is then deposited via a nozzle. Piezoelectric 3D inkjet bioprinters work by applying different voltages to a piezoelectric crystal within the bioprinter. This generates the pressure required to eject the biomaterial containing the cells of interest out of the nozzle [4]. This is commonly used as the process is generally fast and inexpensive [9]. However, most of the problems arise due to clogging of the nozzle, which affects bioprinting precision and droplet direction [10]. Laser-assisted bioprinting or laser-induced forward transfer (LIFT) transfers the energy from a laser beam to a ribbon, which in turn deposits the cell-containing biomaterial onto a receiving substrate [4, 11, 12]. This mechanism overcomes the problems of nozzle clogging and permits high resolution bioprinting [12, 13]. Microextrusion bioprinting works by robotically dispensing cell-containing biomaterials with the aid of mechanical (piston or screw) or pneumatic systems [14–16]. Although microextrusion bioprinting may appear primitive with low resolution, it has a significant advantage of being able to deposit high density biomaterial with high viability [15]. MPE-based bioprinting boasts high resolution by cross-linking polymers and proteins using photo energy [8, 17]. In addition, newer techniques, combinations, and commercial variants have also emerged in recent years [18, 19]. Depending on the type of 3D bioprinting methods used, high resolution bioprinting can achieve resolutions less than 100 nm [20].

## 3. 3D Bioprinting Materials

### 3.1. Scaffold-Dependent 3D Bioprinting

Scaffold-dependent [18] 3D bioprinting of myocardial tissue is more common and requires the incorporation of biomaterials, in the form of scaffolds or bioink [21–23]. The biomaterials utilized to 3D bioprint myocardial tissue include alginate, collagen, gelatin, hyaluronic acid, and decellularized extracellular matrix scaffolds (Table 1).

In the late 1990s, Eschenhagen and colleague successfully fabricated contractile myocardium made of chick embryonic cardiomyocytes, in a collagen matrix [31]. Since then most of the work has been centred on scaffold-dependent 3D bioprinting. However, there are limitations with the utilization of biomaterials, such as immunogenicity to the scaffold, degeneration of biomaterials, toxicity caused by the degraded products, and the additional costs in procuring the biomaterials [32].

#### 3.1.1. Alginate

Alginate hydrogels have demonstrated high applicability as scaffolds for 3D printing [33]. Alginate is notable in its ability to make hydrogels at* in vivo* physiologic conditions, demonstrating therapeutic viability. In addition, alginate's ability to form a gel pore network that allows diffusion of nutrients and waste materials is vital for the functionality of bioprinted myocardium to function like native myocardium. Finally, given its extensive use in tissue engineering, alginate culturing methods are well characterized and reproducible for clinical evaluation.

Bioprinting of myocardium with a scaffold made from alginate was described by Gaetani et al. [24], who used the method of pressure-based extrusion to bioprint a patch of tissue that demonstrated cardiogenic potential. The group used human cardiac-derived cardiomyocyte progenitor cells (hCMPCs) to construct the patch, which demonstrated viability after both one day and one week of culture. In addition, cells bioprinted in the cardiogenic patch demonstrated the ability to migrate from the alginate scaffold and form tubular-like structures, indicating the potential use of this method for therapeutic cell delivery, while retaining functional properties of cardiomyocytes.

#### 3.1.2. Collagen

Jakab et al. [27] demonstrated the use of a “biopaper” hydrogel scaffold composed of rat-tail collagen type 1, on which spheroids of leghorn chicken atrioventricular cells (cAV cells) and human vascular endothelial cells (HUVECs) were printed. The bioprinted spheroid-biopaper tissue successfully fused and demonstrated synchronous beating at 90 hours similar to that of the native chicken cardiomyocytes. In addition, at 90 hours, there was evidence of early vascularization, with regions of elongated endothelial cells that formed vessel-conduit-like structures, signs of long-term viability of this tissue.

The biopaper hydrogel used in this printing method provided the appropriate environment for the spheroids printed on it to fuse and form functional tissue. Jakab et al. note that the biopaper was soft enough to allow the smooth deposition of the spheroids and also provided the appropriate environment for fusion and cell movement. These elements allowed the tissue to properly assemble into functional myocardium.

#### 3.1.3. Hyaluronic Acid/Gelatin

Gaetani et al. [16] demonstrated the use of another scaffold, hyaluronic acid/gelatin, for tissue bioprinting with hCMPCs. Utilizing a murine model of myocardial infarction, the application of the bioprinted myocardium led to a significant reduction in adverse myocardial remodelling, which is thought to be implicated in the exacerbation of progressive heart failure [34]. Murine hearts that received the bioprinted tissue patch also demonstrated improved cardiac function and long-term engraftment of the hCMPCs.

Hyaluronic acid is a naturally occurring molecule in the ECM and thus has long been used as a scaffold for tissue engineering [35]. It is notable for its tensile strength as a biomaterial, which is important in beating myocardium to resist postengraftment destruction. The hyaluronic acid/gelatin complex is attractive due to its demonstrated safety in native tissue when implanted [36].

#### 3.1.4. Decellularized Extracellular Matrix

Cell seeding onto decellularized extracellular matrix from cardiac tissue is an established technique to grow myocardium in vitro. Oberwallner et al. [37] demonstrated the ability to decellularize human myocardium to yield heart decellularized extracellular matrix (hdECM). Tissue retrieved from patients undergoing implantation of a ventricular assist device was decellularized using a detergent method and then incubated in fetal bovine serum, yielding hdECM with native-like levels of collagen, laminin, and fibronectin. Human mesenchymal stem cells, murine cardiomyocytes derived from induced pluripotent stem cells, and naive neonatal mouse cardiomyocytes were seeded on the human hdECM and demonstrated greater proliferation and viability compared to standard culture. Cells seeded on to the human hdECM beat synchronously. However, the use of hdECM is just beginning to flourish.

Pati et al. [25, 38] demonstrated the use of decellularized extracellular matrix as a bioink for 3D bioprinting. In their experiment, porcine heart extracellular matrix was decellularized by a series of biochemical processes to yield hdECM bioink. The resulting hdECM had near-native levels of collagen and glycosaminoglycans, providing a viable environment for 3D bioprinting. A printing method referred to as the multihead tissue/organ building system [39] was used to simultaneously print the hdECM bioink with rat myoblasts to produce a tissue block that demonstrated viability at 24 hours. Gene and protein expression of the myoblasts printed with the bioink demonstrated vital components for graft survival and function. The microarchitecture of the graft also demonstrated native-like organization, suggesting the potential for these grafts to be used for myocardial reconstruction [25].

Jang et al. [40] have since demonstrated a novel technique of producing hdECM complexed with ultraviolet radiation-treated vitamin B2 (VB2). The resulting bioink, printed with human cardiac progenitor cells, was characterized by stiffness similar to native myocardium. The improved tissue stiffness is thought to be the result of VB2, which forms stable cross-links when exposed to ultraviolet radiation. Furthermore, the cardiac progenitor cells actively proliferated and showed signs of effective differentiation.

The extracellular matrix microenvironment is thought to be critical in promoting and guiding stem cell differentiation [38, 40, 41]. The various proteins within the hdECM allowed for the cells bioprinted with this bioink to differentiate and organize with native-like instructions. Furthermore, the bioink provided a structure that supported the bioprinted grafts' long-term viability. Furthermore, the use of fortifying materials such as VB2 may represent approaches to better supporting bioprinted cells on hdECM that closely resembles native ECM [40].

### 3.2. Scaffold-Free 3D Bioprinting

Despite the successes of the experiments detailed so far, the use of scaffolds is not without issues. Scaffolds face the issue of rapid degeneration leading to limited mechanical/physical stability [26, 42]. Investigations into scaffold-free 3D bioprinting of myocardium have yielded some positive results.

Atmanli and Domian [26] demonstrated the novel use of microcontact printing to bioprint functional cardiac tissue that preserves the unique native architecture of myocardium. They did so using double transgenic murine committed ventricular progenitors (CVPs). CVPs resemble cardiac myocytes on a number of parameters including expression of myocardial markers, as well as exhibiting functional and structural properties [43]. Normally scaffolds provide extracellular cues for cardiac progenitor cells to organize into myocytes. In Atmanli and Domian's microcontact printing method, they addressed the need of a scaffold by relying on microtextured polydimethylsiloxane (PDMS) stamps instead, to guide the microarchitecture of the bioprinted tissue. The resulting tissue represented the characteristic anisotropy of native myocardium, which is critical for its mechanical and electrophysiological properties [44–46]. This experiment did not test the* in vivo* therapeutic capacity of myocardium bioprinted in this manner but nonetheless represents a pivotal early step in scaffold-free 3D bioprinting of myocardial tissue.

Tissue-spheroid-based organ bioprinting is emerging as a major alternative to traditional scaffold-based tissue bioprinting [47]. Tissue spheroids are three-dimensional, organized clusters of cells in bulk. When tissue spheroids are placed near each other, surface tension causes them to fuse into a “living material.” This material demonstrates the physiological properties of the native tissue the spheroid cells were derived from. Spheroids provide several advantages in their ability to be organized at a cellular level like organoids in a native tissue, as well as the ability to seamlessly assemble spheroids into larger structures that still preserve the characteristics of the native tissue without the constraints of scaffolds [18]. Tissue spheroids composed of rat neonatal ventricular cardiomyocytes, human dermal fibroblasts, and human coronary microartery endothelial cells were used to form a contractile cardiac patch that remained viable after being transplanted into rats by Noguchi et al. [48]. This demonstrates the potential for myocardium to be constructed using tissue spheroids.

Recent technological advancements have led to 3D bioprinters capable of assembling spheroids, with precise positioning of individual spheroids [18]. This method uses a robotic arm to pick up preassembled tissue spheroids using vacuum suction and impale them onto microneedles arranged in an array. The spheroids subsequently fuse and functional tissues are then removed from the needle arrays for further maturation. This technique offers a reproducible, reliable, and precise technique to bioprint tissue spheroids into organized tissue.

Ong et al. [29, 30] used this method to bioprint tissue spheroids composed of human induced pluripotent stem cell-derived cardiomyocytes (hiPSC-CMs), fibroblasts, and endothelial cells into myocardial patches. The cardiac patches demonstrated spontaneous beating, as well as ventricular-like action potential waveforms and uniform electrical conduction throughout the patches. These patches were then implanted* in vivo* into a rodent model and demonstrated engraftment and vascularization, suggesting the therapeutic regenerative potential of this scaffold-free 3D bioprinting method.

## 4. Cell Types

Cells used in these experiments can be broadly classified into nonhuman and human cells. Some methods used cell mixtures from both types as well. Rat myoblast cells (L6 cell line) have been shown to differentiate into functioning cardiac tissue in a variety of experiments [25, 49]. In Jakab et al.'s [27] study, embryonic myocardium was harvested from leghorn chickens and cardiomyocytes were bioprinted on a biopaper hydrogel. Murine committed ventricular progenitors were used in Atmanli and Domian's scaffold-free method, due to the cells' similarity to human cardiomyocytes by a number of parameters [26, 43].

In terms of human cells [50], hCMPCs and hiPSC-CMs are popular choices for 3D bioprinting [16, 17, 24, 26, 29, 30]. These cells exhibit genetic profiles and express proteins that are vital for differentiation into functional myocardium, and they demonstrate the characteristics of native myocardium when bioprinted in the methods described above.

Mature cardiomyocytes are the result of differentiation of human cardiomyocyte progenitor cells produced from human induced pluripotent stem cells or human embryonic stem cells [51]. Various protocols have been established to differentiate human pluripotent stem cells into beating cardiomyocytes [52, 53], and various groups have demonstrated the therapeutic potential in the repair of damaged myocardium and cardiac regeneration [54–56].

However, immaturity of stem cell-derived cardiomyocytes, due to incomplete maturation [57], remains a major obstacle and promoting cardiomyocyte maturation is important in order to achieve the final goal of cardiac regeneration [58]. Chong et al. [59] observed in a nonhuman primate model of myocardial ischemia-reperfusion that treatment with human embryonic stem cell-derived cardiomyocytes (hESC-CMs) led to significant remuscularization, albeit with nonfatal ventricular arrhythmias, due to incomplete maturation of hESC-CMs. In addition, other issues regarding tumorigenesis, graft viability, and immunological rejection [60] have to be addressed as well, before human studies can be conducted.

3D printing scaffolds separately and then seeding cells have been a major area of research in myocardial tissue engineering as well, though there are significant challenges associated with this method including its resource- and time-intensive requirements [25]. Certain novel methods in 3D printed scaffolds with subsequent cell seeding have yielded viable myocardium [12, 17, 19] with the use of C2C12 myoblast cells, human mesenchymal stem cells (hMSCs), and hiPSC-CMs as the seeded cells. Reviewing cell types that have been seeded into 3D printed scaffolds and the results of these experiments can give new ideas regarding potential cell types for 3D bioprinting.

## 5. Cardiac Cellular Composition

A clear understanding of the myocardial cellular composition is crucial in bioengineering of the myocardium. The specific cellular composition of cardiomyocytes and noncardiomyocytes in the native human myocardium has been a subject of debate [61, 62]. Previously, studies found that fibroblasts represented the majority of noncardiomyocytes but modern techniques in recent literature have showed this may not be true [63–65]. Some of the reasons to explain this discrepancy include utilization of nonspecific markers and reagents, estimation used in stereological approaches, failure to completely dissociate cells, and low cell viability during flow cytometry. These factors can significantly alter the overall distribution. Most recently, Pinto et al. argued that the majority of noncardiomyocytes are endothelial cells [65]. Using improved cell isolation techniques and flow cytometry, they found that more than 60% of the noncardiomyocyte cell population consisting of endothelial cells and fibroblasts contributed to less than 20%, while the remaining cell types included leucocytes, pericytes, and other resident mesenchymal cells. Subsequently, analysis of the human heart also demonstrated predominance of endothelial cells, corresponding to histological studies of the cardiac myocardium conducted much earlier, decades ago [66–68].

Variations in the cell composition implemented during the fabrication of non-3D printed engineered heart tissue (EHT) influenced the structure and function of the cardiac patch [69–72]. Functionally, forces generated by the EHT made from pure cardiomyocytes were three time less than those made from a mix of native cardiomyocytes and noncardiomyocytes [72]. Structurally, the addition of fibroblasts to cardiomyocytes improved cellular architecture with a more uniformed layout [71]. Increased microvascularization was observed with the addition of other cells, such as endothelial cells, fibroblasts, stromal cells, and mesenchymal cells [69, 70, 73]. EHT supplemented with noncardiomyocytes performed better than EHT made of pure cardiomyocytes when implanted* in vivo* [69, 70]. Tulloch and colleagues demonstrated perfusion by host erythrocytes as early as 1 week in implanted EHTs which were supplemented with human umbilical vein endothelial cells, human marrow stromal cells, and mouse embryonic fibroblasts [69].

Likewise, encouraging results were reported in the 3D bioprinting of the myocardium when a mixture of cardiomyocytes and noncardiomyocytes were utilized [12, 17]. Gaebel and colleagues [12] seeded a cardiac tissue patch with HUVECs and hMSCs in a systemic pattern. This was based on evidence that suggested hMSCs could ameliorate angiogenesis in postinfarcted myocardium, affecting cell repair and regeneration processes. They were perceived to be able to prevent apoptosis induced by hypoxic endothelial cells, which in turn promoted angiogenesis and cell survival [74]. Gao and colleagues [17] seeded their native-like ECM scaffold with cardiomyocytes, endothelial cells, and smooth muscle cells derived from human cardiac lineage induced pluripotent cells with good effect. Synchronous contractility was observed on their cardiac patch 1 day after seeding and by day 7 demonstrated good electrophysical properties of cardiac muscle function and the presence of the genetic expressions necessary for contractility.

## 6. Viability and Vascularization

For 3D bioprinting of the myocardium to be feasible in clinical application, tissue perfusion and vascularization are vital. Adequate blood supply to the transplanted 3D bioprinted myocardial graft is important for the long-term durability and viability of the graft, after* in vivo *implantation. For meaningful clinical applications, the myocardial tissues constructed have to be of a reasonable size, and due to this, diffusion alone is usually insufficient to maintain the delivery of oxygen and nutrients necessary for cell survival. To achieve optimal oxygen and nutrient delivery* in vivo*, living cells have to be within 100–200 *μ*m from its supplying capillaries [75].

### 6.1. Vascularization Strategies

There are a number of vascularization strategies that have been studied with varying degree of success, which could be relevant to engineer an organized vasculature within the 3D bioprinted myocardium in a hybrid fashion. Vascularization via cell to cell interaction relies on utilizing proangiogenic cells that are cocultured to enhance vascularization [76]. The addition of endothelial cells* in vitro* during cell culture have demonstrated improved growth and stabilization of vascular networks in engineered tissues, such as skin and skeletal muscle when implanted* in vivo* [76, 77]. Other studies have shown useful application of angiogenic factors, such as vascular endothelial growth factor (VEGF), stromal cell-derived factor 1 (SDF1), or angiopoietin-1 to hasten and promote the formation of vessel networks [77–79]. In essence, endothelial cells are cultured on polyethylene glycol hydrogels with covalently bound angiogenic factors. The bioactive ligands in the hydrogel release the angiogenic factors to direct vessel formation [80]. Capillary like networks were observed as early as two days [81]. Schesny and colleague demonstrated preserved rodent heart function postinfarction with controlled release of SDF1 [79]. Improved regeneration and cell survival in ischemic murine hindlimbs of murine model were reported with the utilization of VEGF releasing hydrogels seeded with endothelial cells [82]. SDF1 has also been shown to enhance neovascularization in ovine hearts postmyocardial infarction [83]. However, these techniques allow limited control over the exact architectural alignment and specifications of the resultant vascular networks formed.

In order to fabricate more precise vascular networks that resemble a native vascular tree, vascularization with the aid of sacrificial materials had been investigated and had shown promise [84–86]. Miller and colleague were able to produce a 3D printed vascular network lined with endothelial cells, using carbohydrate filaments as sacrificial material, which were robust enough to be perfused with pulsatile and high-pressure flows [87]. Another construct using micropores proved successful in sustaining perfusion and thereby maintaining the metabolic processes in a three-dimensionally engineered hepatocyte tissue [85]. Similarly, spatial micropatterning is another useful technique for the fabrication of an organized micro- and nanovasculature. Culver and colleagues were able to use photon lasers to create three-dimensional pathways in a hydrogel lined with angiogenic factors, guiding the growth of endothelial cells into an intricate vascular network [88]. This laser guided cell patterning for the formation of tubular networks within collagen hydrogels was also demonstrated by Hribar and colleagues [89]. Ultrasound standing wave fields are a noninvasive spatial micropatterning technique that directs endothelial cells to form vascular networks within engineered tissues, by controlling acoustics in a sound field [90–93]. Overall, these methods provide hope for further research to better engineer viable vessel networks, enabling prompt and adequate perfusion of implanted tissues in vivo.

### 6.2. Vascularizing 3D Bioprinted Myocardium

Most the work with regard to myocardial vascularization has been initially conducted using non-3D printed EHT in animal models [69, 94, 95]. Rapid angiogenesis of implanted EHT has been previously demonstrated, with a dense network of microvascularization shown within 2 weeks of EHT implantation [69, 95] and host erythrocytes identified in these vessels [70]. Utilizing immunostaining with human-specific CD31 and *α*-smooth muscle actin, Lesman and colleagues demonstrated the origins of both human (donor) and rat (host) vasculature in the human EHT grafts that were implanted in murine hearts [70].

The construction of thick viable myocardial tissue is another challenge. Shimizu and colleagues [96] initially tried to implant cell sheets thicker than 3 layers in one sitting, but due to a lack of intrinsic microvasculature, the implanted tissue quickly became necrotic. Shimizu et al. subsequently managed to construct thick viable myocardium by multiple implantations of thin cell sheets at intervals of 1 to 2 days. The macrovasculature is also important to perfuse thick myocardial tissue grafts. Several innovative designs to address this issue have shown promising results [97–100]. Essentially, the concept involves culturing EHT with arterial and venous vessels in a medium, to promote angiogenesis between the micro- and macrovasculatures. The addition of vascular endothelial cells has also been shown to promote vascularization in 3D bioprinted cardiac constructs, such as in experiments by Jakab et al. [27, 28].

3D bioprinted myocardium has demonstrated vascularization and significant therapeutic effects, when implanted into animal hearts. Gaebel and colleagues demonstrated the presence of primitive vascular networks in 3D bioprinted myocardium constructed by LIFT 3D bioprinting that were seeded with human mesenchymal cells and HUVECs [12]. Eight weeks after infarction, fluorescent-labelled tracings revealed primitive vascular structures, high density of capillary networks, integration with host vasculature, and significant improvement in cardiac function. Similarly, Gaetani and colleagues also reported improvements in cardiac function, after applying 3D bioprinted hyaluronic acid/gelatin patches containing cardiac progenitor cells in postmyocardial infarction murine hearts [16]. These patches showed continued proliferation and maturation when analyzed at 4 weeks, suggesting durability and viability of 3D bioprinted cardiac patches [16]. Gao and colleagues observed increased population of arterioles in the cardiac patches constructed by MPE-based 3D bioprinting after implantation into murine hearts [17]. Contractility was observed from day 1. At 4 weeks, significant therapeutic effects including reduction of infarct size and improved ejection fraction were noted. Our group also constructed scaffold-free 3D bioprinted myocardium and demonstrated viability, vascularization, and engraftment 1 week after implantation [29, 30]. To surmise, multiple* in vivo* studies have demonstrated vascularization of engineered cardiac tissue and the addition of vascular cells* in vitro* may prove beneficial in 3D bioprinting of the myocardium in promoting viability, paving the way for possible clinical application in the future.

## 7. 4D Bioprinting

4D bioprinting incorporates the fourth dimension of “time” into 3D bioprinting. This includes instances in which objects can change their shape based on the presence of external stimuli, such as when cell fusion or self-assembly occurs [101]. With the aim of creating* in vitro *models that resemble tissue structures found in nature, 4D bioprinting promotes dynamic, structural, and cellular changes of a tissue over time, overcoming the static nature of 3D bioprinting [102].

As a new field, the classification and definition of 4D bioprinting are still not universally agreed upon. One classification proposed by An et al. defines 4D bioprinting to include several approaches; two of these approaches include the folding of tissue on a substrate into a desired shape and the self-assembly or self-organization of a tissue. To constitute 4D bioprinting, these transformations must be through the induction of a stimulus, rather than a change caused by natural processes [103]. Although the field of 4D bioprinting of the myocardium is still nascent, there is published literature that provides insights.

The first approach to 4D bioprinting prints tissue onto a substrate material (like a responsive hydrogel) and folds the tissue into a desired shape upon stimulus induction [103]. A number of researchers have worked on creating these shape-morphing substrates and scaffolds: Kirillova et al. experimented with printing shape-morphing hydrogel made of two different biopolymers (alginate and hyaluronic acid) to fabricate hollow self-folding tubes. These formed diameters comparable to those of the smallest blood vessels and maintained the viability of the printed cells for 7 days without a decrease in cell viability [104]. Apsite et al. created electrospun, porous multilayer scaffolds based on thermoresponsive polymers. At different temperatures in an aqueous environment, these scaffolds spontaneously rolled to form a tube with distinct layers. By adding collagen, cell viability and adhesion were improved [105]. Furthermore, self-healing hydrogels have been studied for therapeutic and biomedical applications. These hydrogels increase the lifetime of a functional material and also exhibit similar characteristics to human tissue [106]. Cardiomyocytes cultured in a 3D fibrin hydrogel environment that mimics the cell's natural environment were noted to remodel the construct to generate contractile force under electrical pacing conditions [107]. Culturing cardiomyocytes in a shape-morphing hydrogel can potentially aid the formation of more native-like 3D structures over time. With advances in bioprinting stimuli-responsive hydrogels and cardiomyocyte viability in hydrogels, further advances in this approach of 4D bioprinting can promote the ability to control the spatial arrangement of cells and internal structure of engineered myocardium.

The second approach is to promote the self-assembly of tissues through the stimulation of a printed structure. For example, printed microdroplets can precisely deposit cells in a particular pattern that will conform to another pattern upon stimulation [103]. There has been research that addressed the stimulation of cardiac tissue structures: Kaji et al. [108] analyzed the response to chemical stimuli on a single-cell level. With chemical stimuli, the myocytes conjugated to form gap junctions. A stimulus, caffeine, activates the gap junction communication whereas 1-octanol reversibly inhibits it. Our group has also used defined chemical factors to stimulate scaffold-free 3D bioprinted cardiac patches, to promote better tissue organization and maturity. Serpooshan et al. utilized Faraday waves as a stimulus to enable the aggregation of hiPSC-CMs into predefined 3D constructs [109]. With the ability to 3D bioprint cardiac tissue and a variety of stimulus mechanisms to induce tissue structural and cellular changes over time, 4D bioprinting of the myocardium remains an active area of research, and the considerable possibilities and potentially therapeutic benefits mean that novel work in this field will be highly impactful.

## 8. Challenges and Future Prospects

The choice of 3D bioprinting method and the decision whether to use scaffolds (and if so, which scaffold) are important considerations. During the delivery of the scaffold, cells are sometimes subjected to the unavoidable effects of thermal or vibrational energy and sheering forces, depending on the method of 3D bioprinting. Immunogenicity of the scaffolds is still an issue that has to be addressed. Further studies are needed to evaluate both the short and long-term effects of the by-products released during the breakdown of the scaffolds. Also, most scaffolds generated do not reflect the native architecture of adult myocardium. Decellularization of heart tissue can be a solution as it can create constructs that closely mimic native adult myocardium [110]. On the other hand, most scaffolds are notably porous in nature. This allows waste exchange and vascularization, which aid in graft survival in vivo. In addition, this porosity allows cell attachment and facilitates proliferation and maturation. Scaffolds also boast good tensile strength and the ability for the 3D bioprinting myocardium to withstand high pressure and the forces of myocardial contractions. 3D bioprinting using tissue spheroids is an attractive alternative as it allows customized tissue design without the use of scaffolds and avoids the problems with cell injury during the dispensing of the biomaterial. The production of a desired composition tissue spheroid is relatively straight forward, and it has shown to form cell-cell junctions spontaneously, demonstrating synchronous contraction and the development of primitive vascular like structures [27, 111, 112]. Overall, scaffold-free and scaffold-dependent options have their own advantages and drawbacks, with both showing evidence of continued progress and optimization.

Achieving the ideal cellular composition for cardiac constructs is still challenging, as we are still learning about the native human myocardium. This is complicated by the observations that altering the composition of noncardiomyocytes, such as fibroblasts and endothelial cells, affects the function, vascularization, and viability of the 3D bioprinted myocardium. Ultimately, there may not be a need for complete biomimicry for cardiac regeneration in terms of cell ratio of the cardiac construct, and further studies are needed to investigate this.

Regarding vascularization, significant progress has been reported. Early results from studies incorporating vascular cells into these models are encouraging, with therapeutic efficacy demonstrated in murine hearts. To construct a sizable, macroscale [113], mechanically robust and clinically meaningful 3D bioprinted myocardium, an established intrinsic vasculature for adequate perfusion is necessary to keep up with the high metabolic demands of contractility. Vascularization and scalability represent some of the greatest challenges for the progress of this technology. In addition, further testing in large animal models to evaluate the safety and therapeutic effects of these tissues is needed to provide preclinical data for possible future clinical applications. These results will significantly influence the future of this technology.

We have also listed potential avenues for time-varying 4D bioprinting to transform cardiac tissue engineering. 4D bioprinting utilizes stimuli-responsive materials and shape-memory polymers to create dynamic 3D patterned biological structures that transform in shape and structure, in the presence of a stimulus [104]. Due to the early nature of 4D bioprinting of tissues, it is feasible that new approaches and technologies will emerge once more research is conducted. One possible limitation of 4D bioprinting is the presence of a stimulus, which may present the opportunity to damage and kill living cells. The stimulus must thus be tuned or titrated to ensure that this does not happen to a significant degree.

Overall, 4D bioprinting of the myocardium is in its infancy and although we are years, if not decades, away from major clinical application breakthroughs, the technology presents a number of important opportunities. First, by adding the time dimension to 3D bioprinted materials, 4D bioprinting offers the opportunity to build dynamic structures that better represent the types of tissue structures* in vivo*, for tissue regeneration. Furthermore, by designing structures that can respond to stimuli, we can build cardiac tissue models that respond to unique cell conditions and open their applicability to a variety of treatments.

In conclusion, the evidence presented in this review gives hope for the future. Continued progress will require close collaboration among physicians, scientists, biomedical engineers, and experts from other fields, to optimize this approach combining traditional cardiac tissue engineering techniques with 3D and 4D bioprinting.

## Figures and Tables

**Table 1:** Examples of 3D bioprinting of the myocardium: methods, cell types, and scaffolds used.

3D bioprinting method	Cell type used	Scaffold	Reference
Pressure based extrusion	hCMPCs	Alginate	Gaetani et al. [24]

Multihead tissue/organ building system	Rat myoblast cells	Decellularized extracellular matrix	Pati et al. [25]

Tissue printing	hCMPCs	Hyaluronic acid/gelatin	Gaetani et al. [16]

Microcontact printing	Double transgenic embryonic stem cells (mouse embryonic fibroblast + embryonic stem cell)	Scaffold free	Atmanli and Domian [26]

Spheroid extrusion	Leghorn chicken atrioventricular cells, human vascular endothelial cells	Collagen	Jakab et al. [27, 28]

Spheroid bioassembly	hiPSC-CMs	Scaffold free	Ong et al. [29, 30]
